# First Report of Single‐Surgeon Single‐Incision Laparoscopic Percutaneous Extraperitoneal Closure Using the Senhance Robotic System as Camera Holder for Pediatric Inguinal Hernia

**DOI:** 10.1111/ases.70131

**Published:** 2025-08-13

**Authors:** Daiki Kato, Takahisa Tainaka, Chiyoe Shirota, Satoshi Makita, Katsuhiro Ogawa, Masamune Okamoto, Akihiro Yasui, Shunya Takada, Kaito Hayashi, Yoichi Nakagawa, Hiroki Ishii, Hajime Asai, Ami Utsunomiya, Akinari Hinoki, Naoki Nagata, Hiroo Uchida

**Affiliations:** ^1^ Department of Pediatric Surgery Nagoya University Graduate School of Medicine Nagoya Japan; ^2^ Department of Gastrointestinal Surgery Kitakyushu General Hospital Fukuoka Japan

**Keywords:** child, inguinal hernia, laparoscopic percutaneous extraperitoneal closure, robotic surgical procedures, single incision

## Abstract

Single‐incision laparoscopic percutaneous extraperitoneal closure (SILPEC) is used for pediatric inguinal hernia repair in several institutions. However, SILPEC requires at least two operators. We report the first SILPEC performed by a single surgeon using the Senhance robotic system for pediatric patients. The Senhance, which features reusable 3‐ to 5‐mm instruments and tremor filtering, allows both eye‐tracked and manually adjustable camera control via a standalone robotic arm, resulting in stable, assistant‐free visualization while preserving the small port philosophy. Two pediatric patients with inguinal hernia underwent SILPEC. The laparoscope was mounted on one robotic arm, and a 3‐mm curved grasper was manipulated manually through the same incision. Operative times were 71 and 77 min, without blood loss, conversions, or perioperative complications. Both patients remained recurrence‐free. This report demonstrates the feasibility, safety, procedural efficiency, and potential cost‐effectiveness of single‐surgeon robotic assisted SILPEC, thus offering a pragmatic solution for understaffed pediatric surgical units.

## 1 Introduction

Inguinal hernia is one of the most common conditions treated by pediatric surgeons, and surgical repair is among the most frequently performed procedures by pediatric surgeons worldwide. Laparoscopic percutaneous extraperitoneal closure (LPEC) has gained widespread acceptance in Japan because it is a simple, safe, and cosmetically favorable technique [1]. Our group further developed single‐incision LPEC (SILPEC) to reduce the number of incisions and improve esthetic outcomes [2] and previously reported its safety and efficacy [3, 4].

Robotic surgery has rapidly expanded recently, and its adoption across pediatric surgical procedures has increased. The Senhance robotic system (Senhance; Asensus Surgical Inc., Naderhan, NC, USA), which was first reported in 2012 [5]. Senhance comprises separate movable robotic arms (between one and four) and a separate console [6]. A laparoscope or forceps can be attached to a robotic arm via a magnetic adapter for quick attachment and removal during an emergency. This system enables camera control through an eye tracker and allows easy manual adjustment of the arm directly in the surgical field. Another advantage of this system is its port compatibility; conventional 3‐, 5‐, and 12‐mm trocars can be used without modification. The surgeon operates the forceps using a sensor‐equipped handle, similar to that used during standard laparoscopic or thoracoscopic surgery, with tremor elimination and haptic feedback. Because of these features, Senhance is suitable for delicate pediatric procedures [6, 7, 8]. Although previous reports have described robotic‐assisted pediatric inguinal hernia repair using Senhance [9, 10], these procedures involved multiple ports, multiple surgeons and assistants, and did not include SILPEC. This report describes SILPEC performed by a single surgeon using Senhance with a laparoscope mounted on a robotic arm. In Japan, where pediatric surgical departments often operate with limited staff, surgeons are required to handle a broad range of clinical responsibilities. Therefore, improving operational efficiency, including the ability to perform procedures independently, has become increasingly important. In this context, robotic systems that support single‐surgeon procedures are particularly promising. To the best of our knowledge, this is the first report of single‐surgeon SILPEC with Senhance for pediatric patients.

## 2 Materials and Methods

In order to operate the Senhance, surgeons must complete a two‐day training program consisting of a dry lab and a written exam to get a certification. After getting a certification, surgeons can immediately begin using the Senhance system. In this study, the surgeon manually adjusted the robotic camera arm rather than using the eye‐tracking function, allowing for intuitive control without a dedicated learning curve for camera navigation.

**FIGURE 1:**
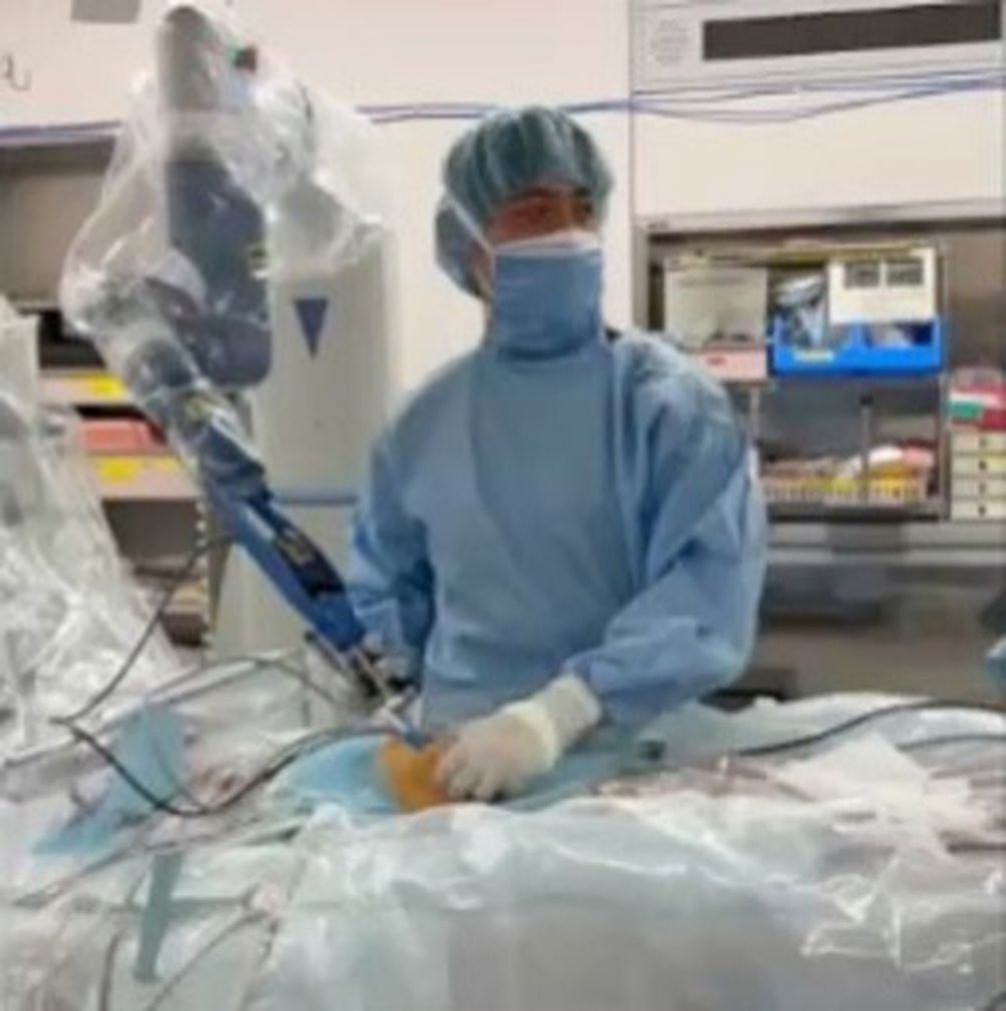
Placement of the surgeon, robot arm, and port. The operator stands on the opposite side of the inguinal hernia and a robot arm equipped with a laparoscope is placed on the same side. A camera port and a grasping forceps are inserted through the same umbilical incision.

**FIGURE 2:**
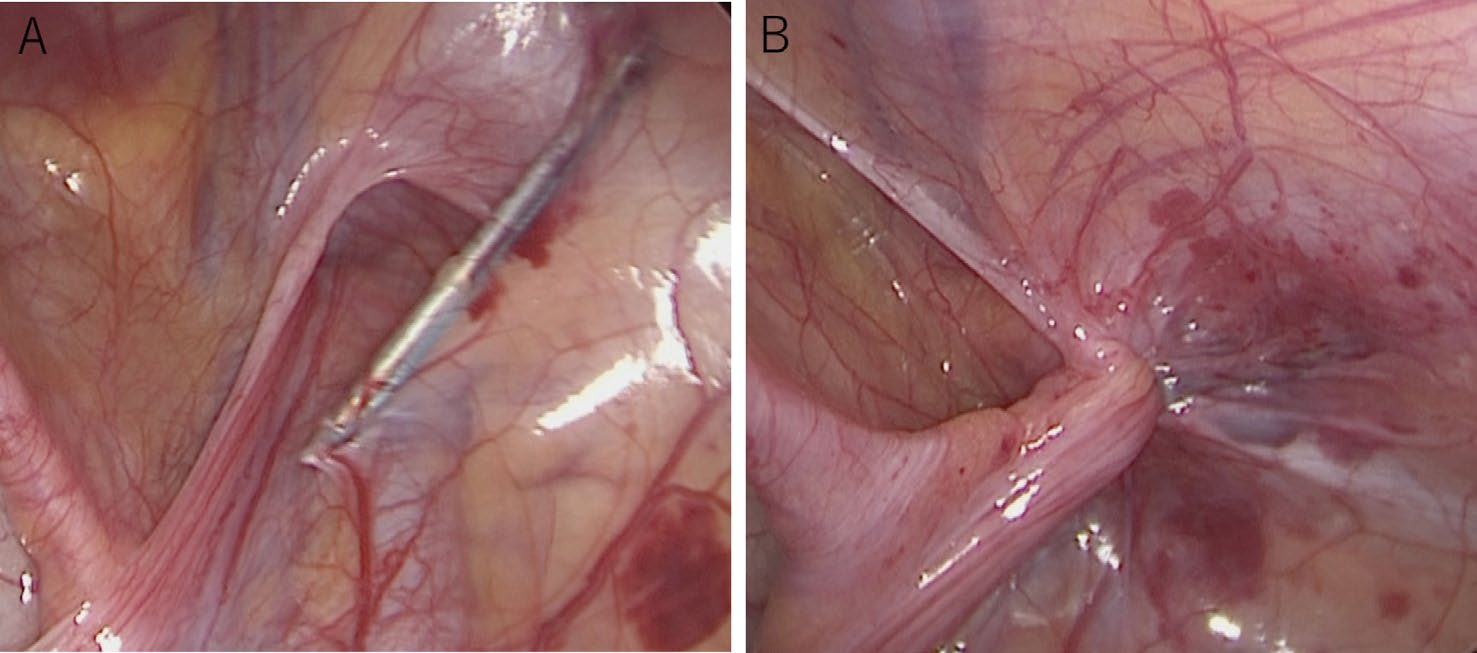
Intraoperative findings. A: Using laparoscopic percutaneous extraperitoneal closure (LPEC) needles and grasping forceps, circumferential sutures are placed around the internal inguinal ring extraperitoneally. B: The circuit suture is tied extracorporeally, and the hernial sac in the inguinal region is completely closed.

The SILPEC procedure has been described previously [2]. The patients were placed in the supine position under general anesthesia. The operator stood on the opposite side of the inguinal hernia, and a robot arm equipped with a laparoscope was placed on the same side (Figure 1). We used a slightly enlarged Mercedes incision at the umbilicus to both provide sufficient working space and allow for cosmetic umbilicoplasty after the procedure. This approach enables the fascia to be adequately exposed while ensuring a clean and natural umbilical appearance postoperatively. A 5‐mm port for a 5‐mm, 30° laparoscope was placed using an open technique, and Senhance was rolled in. The laparoscope was mounted on the robotic arm using a magnetic adapter. The surgeon adjusted the camera position manually by grasping the robotic arm and releasing the clutch, rather than using the eye‐tracking function. This method allowed precise and stable control of the laparoscope to maintain a clear view of the needle tip during suturing. A 3‐mm curved grasping forceps was inserted through the same umbilical incision via a separate fascia entry. A 19‐gauge LPEC needle (LAPA‐HER‐CLOSURE; Hakko, Osaka, Japan) with a 2–0 non‐absorbable suture was inserted at the midpoint of the affected side of the inguinal line. Using the LPEC needle, and with the aid of the grasping forceps, the hernial sac was closed extraperitoneally with circuit suturing around the internal inguinal ring without any peritoneal gap. Circuit suturing was performed extracorporeally, and the hernial sac in the inguinal region was completely closed (Figure 2).

## 3 Case Presentation

### 3.1 Case 1

The patient was a 5‐year‐old girl weighing 18 kg and having a bilateral inguinal hernia. She had no relevant medical history. Swelling in both inguinal lesions was noticed 5 months before presentation to our hospital. She underwent SILPEC with Senhance, with an operative time of 1 h and 11 min and blood loss of 0 mL. The postoperative course was favorable. Two months after surgery, no complications and no recurrence were observed.

### 3.2 Case 2

The patient was a 12‐year‐old boy weighing 44 kg and having a bilateral inguinal hernia. He had no relevant medical history. Swelling in both inguinal lesions was noticed 4 years before presentation to our hospital. He underwent SILPEC using Senhance (Video S1), with an operative time of 1 h and 17 min and blood loss of 0 mL. The postoperative course was favorable. Two months after surgery, no complications and no recurrence were observed.

## 4 Discussion

SILPEC performed by a single surgeon using Senhance was completed without intraoperative complications, conversions, or postoperative issues such as recurrence; thus demonstrating the feasibility and safety of this approach. Laparoscopic surgery, including LPEC, has become increasingly popular for pediatric inguinal hernia repair [11, 12] because it provides reduced invasiveness, excellent surgical visibility, the ability to evaluate and address contralateral hernias simultaneously, and the ability to prevent damage to the vas deferens and spermatic cord vessels. LPEC is one of the simplest and most reliable techniques because of its minimal incisions, low complication rate, low recurrence rate, and enhanced cosmetic outcomes [13]. However, it requires two skin incisions for the laparoscope and grasping forceps. To reduce the number of incisions required and achieve better cosmetic results, we devised a technical method called SILPEC [2], which involves inserting grasping forceps through an entrance that is different from that used for the umbilical port. We have performed numerous SILPEC procedures and reported their safety and efficacy [3, 4].

Previous reports described robotic‐assisted surgery using the daVinci system (da Vinci; Intuitive Surgical Inc., Sunnyvale, CA, USA) or Senhance for pediatric inguinal hernia [10, 14, 15]. However, da Vinci has larger trocars and instruments. Currently, the robot endoscopes available for use with da Vinci are 12 and 8 mm; however, the endoscopes commonly used for pediatric laparoscopic surgery are 3 and 5 mm. Moreover, previous reports of surgeries performed using Senhance described the use of multiple ports. Surgery with small incisions and fewer incision sites is considered appropriate for small children. Particularly, the aforementioned surgery should be performed for inguinal hernias, which are common and benign.

For our patients, we used a robotic arm to hold the laparoscope, thus allowing stable, tremor‐free imaging and surgeon‐controlled camera navigation. Enhanced visual stability is particularly beneficial because it allows the operator to avoid critical structures such as the vas deferens and spermatic vessels in male patients. The elimination of camera shaking and the ability to maintain a consistent surgical view without the need for a human assistant contribute to the safety and precision of the procedure. In Japan, where pediatric surgical departments often operate with limited staff, enabling a single surgeon to perform procedures such as SILPEC is particularly advantageous. This approach allows personnel to be distributed more efficiently across multiple tasks, which is critical in busy clinical settings. The use of Senhance by a single surgeon significantly enhances workflow flexibility and surgical autonomy. However, maintaining a robust support system to promptly address intraoperative emergencies remains essential. At our institution, although SILPEC is performed by a single surgeon, additional trained personnel are always on standby and can be mobilized immediately if necessary. This system ensures that safety is not compromised and operational efficiency is maintained.

In terms of cost, Senhance offers significant advantages. The system supports truly reusable instruments, thus avoiding the high costs of disposable instruments associated with platforms such as the da Vinci system. In Japan, Senhance has been covered by the national insurance for laparoscopic procedures and thoracoscopic surgeries since 2019 and 2022, respectively. Previous studies suggested that Senhance is cost‐effective and associated with procedural expenses that are comparable to or less than those of conventional laparoscopy and substantially less than those of da Vinci [16]. Surgery using Senhance is expected to reduce not only labor costs but also medical expenses. In addition to performing standard robotic procedures, the Senhance system can function independently as a camera holder. Surgeons can easily reposition the camera within the operative field without interference, thus making it user‐friendly and efficient. This versatility further contributes to the system's economic efficiency and practical value in operating rooms.

In conclusion, SILPEC by a single surgeon using Senhance represents a novel, minimally invasive, and potentially cost‐effective approach to pediatric inguinal hernia repair. This technique also offers high procedural efficiency; thus, making it particularly advantageous in healthcare settings with limited surgical staff and an increased demand for productivity.

## Author Contributions

D.K., H.U., C.S., T.T., S.M., K.O., M.O., A.Y., S.T., K.H., Y.N., H.I., H.A., A.U., H.A., and A.H. contributed equally to the conception and design of the study. D.K. and H.U. contributed to the design of the study. A.Y., S.T., K.H., Y.N., H.I., H.A., A.U., H.A., and A.H. contributed to the acquisition and analysis of the data. All authors contributed to data interpretation. D.K., H.U., C.S., T.T., S.M., K.O., and M.O. All authors critically revised the manuscript; agreed to be fully accountable for ensuring its integrity and accuracy; and have read and approved the final version of the manuscript.

## Ethics Statement

All procedures involving human participants were performed in accordance with the ethical standards of the institutional or national research committee and the 1964 Declaration of Helsinki and its later amendments or comparable ethical standards.

## Consent

Written consent to publish this information was obtained from the parents of all study participants.

## Conflicts of Interest

Dr. Hiroo Uchida is an Editorial Board member of *ASES*. To minimize bias, he was excluded from all editorial decision‐making related to the acceptance of this article for publication.

## Supporting information


**Video S1:** Outside view of operative procedure with Senhance for case 2.

The operator stood on the opposite side of the inguinal hernia, and a robot arm equipped with a laparoscope was placed on the same side. A 19‐gauge laparoscopic percutaneous extraperitoneal closure (LPEC) needle and a 2–0 non‐absorbable suture are inserted at the midpoint of the affected side of the inguinal line. Using the LPEC needle and 3‐mm curved grasping forceps, the hernial sac was closed extraperitoneally.

## Data Availability

The data that support the findings of this study are available from the corresponding author upon reasonable request.
